# Telencephalon transcriptome analysis of chronically stressed adult zebrafish

**DOI:** 10.1038/s41598-018-37761-7

**Published:** 2019-02-04

**Authors:** Victoria Huang, Anderson A. Butler, Farah D. Lubin

**Affiliations:** Department of Neurobiology, University of Alabama at Birmingham, Birmingham, AL 35294 USA

## Abstract

Chronic stress leads to disruptions in learning and memory processes. The effects of chronic stress experience on the adult zebrafish brain, particularly the memory associated telencephalon brain region, is unclear. The goal of this study was to identify gene expression changes in the adult zebrafish brain triggered by chronic unpredictable stress. Transcriptome analysis of the telencephalon revealed 155 differentially expressed genes. Of these genes, some are critical genes involved in learning and memory, such as *cdk5* and *chrna7*, indicating effects of chronic unpredictable stress on zebrafish memory. Interestingly, several genes were annotated in the Orange domain, which is an amino acid sequence present in eukaryotic DNA-binding transcription repressors. Furthermore, we identified *hsd11b2*, a cortisol inactivating gene, as chronic stress-responsive in the whole zebrafish brain. Collectively, these findings suggest that memory associated gene expression changes in adult zebrafish telencephalon are affected by chronic stress experience.

## Introduction

Stress experience and anxiety affect physiology, behavior, and brain gene expression across many taxa. Stress response is a short-term adaptation to challenges in the environment, however, when prolonged, leads to detrimental pathologies and learning and memory impairment^1,2^. These health and behavioral consequences are reflected in gene expression changes in the brain, particularly in regions involved in cognitive processes such as the hippocampal region^3,4^.

Like other vertebrates, zebrafish (*Danio rerio*) also exhibit behavioral and gene expression responses to stress and anxiety that allow them to be models for cognitive disorders and dysfunction. Chronic unpredictable stress alters zebrafish swimming behavior and reduces performance in learning tests, increases body cortisol levels and corticotropin releasing factor levels, and alters glucocorticoid receptor (*nr3c1*) gene expression in the whole brain^5–7^. In the zebrafish telencephalon, which includes memory-related brain regions homologous to the mammalian hippocampus and amygdala^8,9^, chronic unpredictable stress can increase mineralocorticoid, and glucocorticoid receptor gene expression as well as thin dendritic spine formation, which are involved in learning and memory^4,6,10^. Chronic stress experience also alters whole brain protein expression, in particular proteins involved in mitochondrial function and neurogenesis^11^.

Zebrafish hypothalamic-pituitary-interrenal (HPI)-related neuronal gene expression has been characterized and elaborated under the influence of chronic stress, but less is known about how this experience influences neuroprotective or learning and memory-associated gene expression. 11-beta-hydroxysteroid dehydrogenase 2 (*hsd11b2*) is neuroprotective in the adult zebrafish brain^12,13^, converting cortisol to inactive cortisone^14^. Yu *et al*.^15^ demonstrated that that adult zebrafish acetylcholinesterase (*ache)* mutants with impaired function have reduced age-associated memory impairment. Acetylcholinesterase breaks down acetylcholine, which is involved in memory formation^16^. While *ache* expression is affected by stress in mice, it has not been examined in zebrafish^17^.

Transcriptomic approaches such as microarrays and RNA-sequencing in the adult zebrafish whole brain have revealed drug-inducible as well as heritable gene expression differences. In the whole brain, *hsd11b2* and anxiolytic gene neuropeptide Y (*npy*) expressions were greater and less, respectively, when treated with anti-anxiety drug fluoxetine^18^. In the telencephalon, administration of pharmaceutical estrogen 17alpha-ethinylestradiol generated a greater *npy* expression^19^. Populations or lines of zebrafish have been selectively bred to maintain consistent behavioral traits such as bold or shy, and stress-reactive or proactive^20,21^, where *hsd11b2* is greater expressed in a stress-reactive vs stress-proactive strain^22^. Taken together, *hsd11b2*, *ache*, *npy* are stress-responsive but have not yet been explicitly addressed in the context of chronic stress. While gene expression changes related to stress and anxiety have been demonstrated, chronic stress-induced transcriptomic changes have not been examined in either the whole brain or telencephalon region.

In this study, we examined the effects of chronic unpredictable stress on adult zebrafish swimming behavior, and the brain from two approaches: (1) selected neuroprotective or memory-related candidate genes in the whole brain (*hsd11b2*, *ache*, *npy, nr3c1*) and (2) unbiased transcriptomic differences in the telencephalon via RNA-sequencing. In the whole brain, reduced *hsd11b2*, and increased *ache* and *nr3c1* mRNA levels were found in zebrafish with chronic stress experience while *npy* was not. We were interested in how stress experience would affect long term memory, 24 hours after the last experience. Telencephalon transcriptome data revealed differentially expressed *cdk5, chrna7*, and *draxin* genes, which is found to alter memory in mouse models^23–25^. In addition, chronic unpredictable stress upregulated genes were significantly enriched for the Orange domain, a transcriptionally repressive family of transcription factors.

## Results

### Novel tank test

Males in the stressed group did not differ from the control group in total distance swam (t = 1.7559, df = 3.8452, p = 0.1568) nor in the time spent at the bottom half of the tank (W = 13, p = 0.2). Among females, chronically stressed vs control groups did not differ in total distance swam (t = −1.4688, df = 9.1621, p = 0.1754) or time spent at the bottom half of the tank (t = 1.2998, df = 9.9709, p = 0.2229) (Supplement 1).

### Cortisol measurement

Trunk cortisol assay showed differences between control and stress group (F = 7.3354, df = 1, p = 0.01039) and among the time of cortisol collection (F = 5.1346, df = 2, p = 0.01108). Benjamini-Hochberg *post hoc* analysis of pairwise t-tests showed trunk cortisol differences between stress vs control groups 15 minutes after the last stress experience (p = 0.050), but not 2 or 24 hours after (p = 0.889, p = 0.117, respectively). Intra-assay variations (%CV) were 2.3 and 2.7, and inter-assay variation was 5.4%.

### Whole brain reverse transcription quantitative PCR

**Figure 1:**
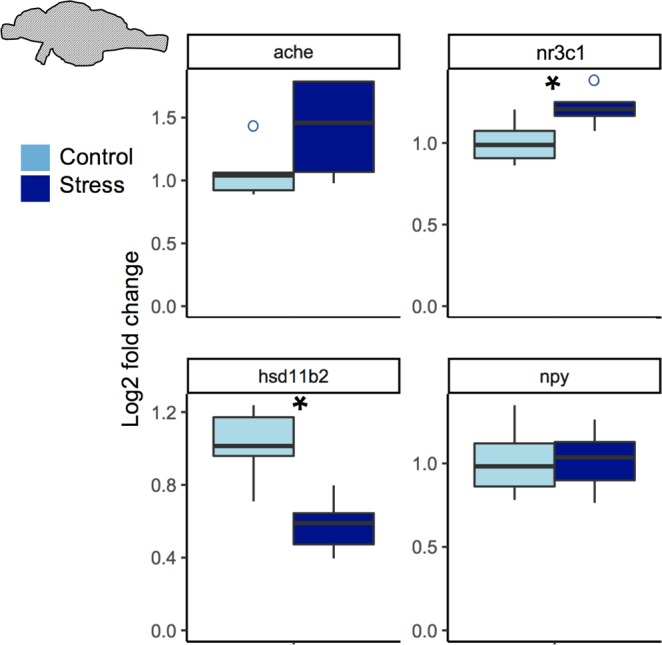
Stress related gene expression in male whole brains. *ache, nr3c1*, *hsd11b2, npy* expression normalized to *ef1a*(elongation factor 1α). Horizontal lines indicate the median. Vertical lines indicate 1.5 times the interquartile range. Open circles indicate values outside the interquartile range. Outliers were not included in analysis. *p < 0.05, n = 5 for control and stress.

**Figure 2:**
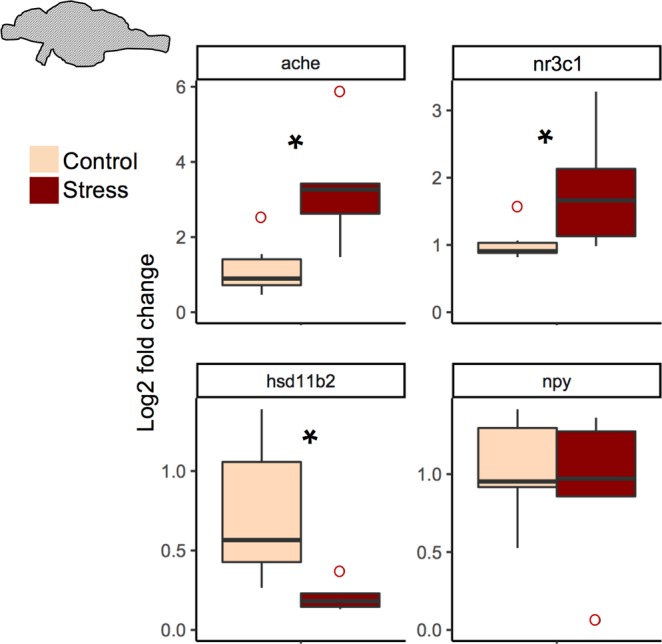
stress related gene expression in female whole brains. *ache, nr3c1*, *hsd11b2, npy* expression normalized to *ef1a* (elongation factor 1α). Horizontal lines indicate the median. Vertical lines indicate 1.5 times the interquartile range. Open circles indicate values outside the interquartile range. Outliers were not included in analysis. *p < 0.05, n = 6 for control and stress.

In males, stressed group had reduced gene expression of *hsd11b2* (t = 3.7848, df = 7.438, p = 0.00612) and a greater mRNA levels of *nr3c1* (t = −2.6231, df = 7.711, p = 0.03148) compared to the control group, but did not have significant fold change in *ache* (t = −1.7615, df = 6.3, p = 0.1263) or *npy* (t = 0.0077585, df = 7.843, p = 0.994) (Fig. 1). The stressed female group had greater *ache* (t = −3.1475, df = 7.5051, p = 0.01481) and *nr3c1* (W = 5, p = 0.04113) mRNA levels compared to control, and a reduced *hsd11b2* expression compared to control (t = 3.2179, df = 6.6168, p = 0.01588) (Fig. 2). However, the stressed females did not show any fold change in *npy* mRNA levels (t = 0.52245, df = 8.4006, p-value = 0.6148). The reference gene *elfa* was not differentially expressed between controlled and expressed for males or females, respectively (t = 0.48193, df = 6.9816, p = 0.6446; t = −0.74604, df = 6.0601, p = 0.4836).

### Telencephalon RNA-sequencing

**Figure 3:**
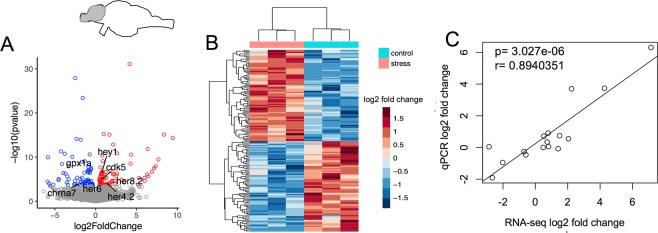
Pooled telencephalon transcriptomic survey. (**A**) Volcano plot of differentially expressed genes in chronically stressed vs. control. Negative and positive log_2_ fold changes with FDR adjusted p < 0.05 after are in blue and red, respectively. (**B**) Heatmap of normalized transformed counts among stressed and controlled samples. (**C**) log2 fold change of selected differentially expressed gene in RT-qPCR and RNAseq.

Of the 24035 genes sequenced (Supplement 2), 155 sequences were differentially expressed between chronically stressed and control group (BH *post hoc* p adjusted < 0.05) (Fig. 3A). Of these genes, 78 had greater counts and 77 had fewer counts in stressed compared to the control group (Fig. 3B).

To validate sequencing results, expression of select genes observed to be differentially expressed via RNAseq was assayed in pooled telencephalon mRNA samples from control and stressed males using reverse transcription quantitative real-time PCR (RT-qPCR). Reference gene *elfa* was not differentially expressed between the two groups. Genes were selected for verification based on previous publications in stress, anxiety, learning and memory (*cdk5*, *chrna7*, *gpx1a*, *jun*, *igf1bp2a*)^21,24^. We also compared expression of additional genes that spanned fold change, and found that the RT-qPCR expression changes were positively correlated to the RNA-seq expression changes (r = 0.8940351, df = 14, p = 3.027e-06) (Fig. 3C).

### Functional annotation of differentially expressed genes

Among downregulated genes, functional enrichment analysis with DAVID detected significant enrichment of the Molecular function GO term ‘structural molecule activity’ (GO:0005198, Fig. 4A), which contained six significantly repressed genes (*cldna, cldnb, evplb, krt4, krt97, and si:dkey-183i3.5;* Benjamini Hochberg adjusted p = 4.1e-3, 14.72 fold enrichment).

**Figure 4:**
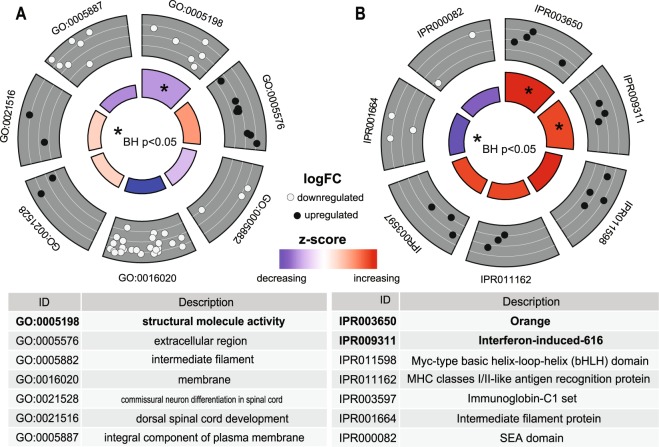
Functional enrichment of telencephalon transcriptome. Enrichment analysis with DAVID revealed a single significantly downregulated GO term and no significantly upregulated GO terms (**A**,**B**), while two Interpro protein domains were significantly upregulated (**C**,**D**). Comparison of GO term annotation for differentially expressed Orange domain-containing proteins reveals a potential role for a subfamily of Orange-related proteins in neurogenesis and chronic stress.

For upregulated genes, functional enrichment analysis with DAVID revealed no significantly enriched GO terms in either the Biological pathways, Cellular components, or Molecular functions categories (Benjamini Hochberg adjusted p > 0.05). However, two enriched protein domains were detected within upregulated genes (Fig. 4B): the Interpro terms ‘Orange’ and ‘Interferon-induced 6–16’ (Benjamini Hochberg adjusted p = 6.4E-4, p = 0.012, respectively). To gain insight into the biological role of differentially expressed Orange domain-coding genes (*her4.2, her6, her8.2, hey1)* across species, we compared GO term annotation for zebrafish and murine homologs of differentially expressed Orange domain-coding genes.

## Discussion

Of the 155 differentially expressed genes in the chronically stressed adult zebrafish telencephalon, cyclin-dependent kinase *cdk5* and nicotinic acetylcholine receptor subunit *chrna7* were identified to have function in learning and memory in mammals^23,26–29^. *Draxin* was also described in rodents to regulate hippocampal organization and neurogenesis, but has not yet been described as stress responsive in zebrafish or mammals^25^. In zebrafish and chronically stressed mammals, *chrna7* is implicated in condition placed preference, a type of Pavlovian conditioning used to measure the motivational effects of experiences^30,31^, and *cdk5* has not yet been described in memory or stress experience in the zebrafish brain. *loxl2b* was also found to be differentially expressed, and its expression is greater after memory tests in the neocortex of mice, though there is no described neocortex homolog in teleosts^32^. Previous gene expression studies on chronically stressed zebrafish telencephalon using RT-qPCR identified differentially expressed glucocorticoid receptor (*nr3c1*)^6^. We did not find this difference in our RNA-seq data, though the previous study chronically stressed zebrafish during the dark cycles that could intensify the stress response. Identifying differential *cdk5, chrna7*, and *draxin* expression in adult zebrafish telencephalon provides an avenue to manipulate learning and memory after stress experience.

In our examination of the telencephalon transcriptome, we identified concerted downregulation of Gene Ontology Biological Process ‘structural molecule activity’ (Fig. 4A). These findings are partially recapitulated by studies in other organisms, which have observed chronic-stress and glucocorticoid receptor-mediated repression of transcription among claudins^33–35^ and in zebrafish, where *cldnb* and *krt4* have previously been identified as a molecular biomarker of stress^36^. In the whole brain, Wong *et al*. had identified the aforementioned GO term differentially expressed between the sexes^37^, but not in an anti-anxiety study nor among strain comparisons^18,22^. It is possible that the whole brain might have a different transcriptome profile, but this comparison has not yet been made.

While our observations of repressed genes are consistent with previous findings, our observations of enrichment in differential upregulation of Orange domain-related genes (her4.2, her6, her8.2, hey1) as well as Interferon alpha-inducible protein IFI6/IFI27-like proteins (IFI6/IFI27-like; si:dkey-188i13.7, zgc:152791, zgc:123068) in the chronically stressed zebrafish telencephalon are novel. The IFI6/IFI27-like protein domain is still very poorly characterized, especially in zebrafish, and has not yet been associated with biological processes or molecular functions; however, the upregulation of genes with this structural domain after chronic stress suggests putative involvement for this in the response to chronic unpredictable stress.

The Orange domain is a ~35aa motif present in eukaryotic DNA-binding transcription repressors. It includes *her6* and, which are induced after injury in the adult zebrafish telencephalon^38^. Interestingly, these genes are both notch effectors and regulators of neurogenesis, which together have been linked to stress in rodent studies^39^. Although evidence for these functions are less well-annotated in zebrafish, some evidence exists for dysregulation of individual rodent homologs as a results of chronic stress^33^, consistent with a putative role for regulation of the Orange domain proteins in the Notch signaling cascade after chronic stress experience.

In both male and female adults, *hsd11b2* levels were lower in chronically stressed vs control groups, which had only been described after an acute stress experience. Because the 11-beta hydroxysteroid dehydrogenase inactivates cortisol, it would be expected that higher levels of *hsd11b2* could mitigate glucocorticoid signaling in the stressed group. However, this lower level of expression was consistent with decreased *hsd11b2* that was found 22–24 hours after acute stress studies in adult zebrafish brain and in the kidney of a common carp strain^12,40^. Alderman and Vijayan^12^ suggested that turnover to protein could decrease *hsd11b2* mRNA levels. Another possibility is that stressed vs control zebrafish exhibited behaviors that induced *hsd11b2* expression; zebrafish that exhibited “shy” behaviors have recorded a greater expression of hypothalamic *hsd11b2* than their bold counterparts^41^. Our finding of lower *hsd11b2* in stressed zebrafish was opposite of the transcriptomic study that found a greater *hsd11b2* expression in control vs. anti-anxiety fluoxetine treated zebrafish whole brains^18^. In the aforementioned study, the tissue collection was taken with, not after the treatment. Additional time- course measurements, as well as hsd11b2 protein assays would further address the possible explanation of gene expression and translation during recovery from stress experiences.

*nr3c1* expression was greater in chronically stressed vs control groups in both males and female adults. This trend was consistent with Pavlidis *et al*.^7^ which saw an increase in the glucocorticoid receptor gene expression but the opposite finding of Piato *et al*.^5^. The former stress experience regiment was 11 days and included light and dark manipulations, while the latter included heated and cold water changes, though the protocol for this study included neither. In Manuel *et al*.^6^ an increase in glucocorticoid receptor expression was not found in the telencephalon after a 14-day chronic stress experience, but was detected after a 7-night chronic stress experience. Together, these results show that the intensity and duration of stress experience modulate glucocorticoid receptor gene expression.

*ache* expression was not found to be different in males but was greater in stressed females than in control females. While there have been no studies on *ache* overexpression in relation to zebrafish learning and memory, mutant zebrafish with reduced *ache* expression have enhanced memory performance^15^. In mammalian hippocampus, acute stress experience affects *ache* expression, and chronic stress decreases acetylcholinesterase activity^17,42^. This study did not quantify the activity of acetylcholinesterase, which can alter learning and memory^16,43^. Though this study did not aim to investigate sex differences, it should be noted that separating sexes can result in varied zebrafish response to chronic unpredictable stress^44^.

Stress experience in this study did not result in any detected differences in expression of the anxiety-related gene *npy* between stress and control groups, which was consistent with no detected differences of anxiety-like swimming behavior in the novel tank: both groups spent most of the novel tank time at the bottom half of the tank. Learning and memory behaviors were not tested, which could be affected by altered *nr3c1* and *ache*^4,15^. While trunk cortisol comparisons from other stress regimens are collected at one time after the last stressor or a behavior test^6,10^, our study was the first to show trunk cortisol levels at multiple time points after the last chronic stressor. We sought to determine if trunk cortisol levels were altered up to 24 hours after the last stressor, when we collected brain tissue for gene expression measurements. While we found no trunk cortisol differences between stress vs control groups after 24 hours, we observed elevation of trunk cortisol 15 min after the last chronic stress experience, showing a physiological response to the stress experience. Animals were sacrificed 24 hours after the completion of the 14 days of chronic unpredictable stress, at a time when trunk cortisol differences were not detected between stress vs control groups, suggesting that long-term changes in RNA expression exist after the cessation of stress hormone elevation. We acknowledge that the various chronic stress paradigms can result in differing physiological responses at 24 hours^45^. Further, as behavioral differences can be detected despite no differences in cortisol or gene expression^6^, it is possible that stress-related gene expression changes may be detected in the absence of differential behavioral or cortisol data.

In summary, transcriptomic differences detected in the telencephalon of chronically stressed adult zebrafish revealed new stress-responsive genes with a putative role in learning and memory, in particular *cdk5*, *chrna7*, *draxin* and genes with the Orange domain (*her4.2, her6, her8.2, hey1*), as previously identified in mammals. We did not detect previously identified telencephalon gene expression differences, which suggests that subtle differences in stressors might impact transcriptome differences. While not differentially expressed in the telencephalon, we identified whole brain *hsd11b2* expression as responsive to chronic unpredictable stress experience. Future studies should include additional behavioral paradigms, such as learning and memory tests, to evaluate the role of identified candidate genes in neurons.

## Materials and Methods

### Animals

Animals were 11 month old male and female adult AB wild type zebrafish (*Danio rerio*) bred at the University of Alabama at Birmingham, Alabama Zebrafish Research Facility (UAB, ZRF). Animals were housed in 3 liter tanks at a density no more than 5 fish/L, in a rack with a closed system filtration of the fish water by Aquaneering (San Diego, CA, USA). Zebrafish were housed on a 14:10 light: dark cycle with water temperature of 28 °C. Zebrafish were fed three times a day with Gemma (Skretting, Westbrook, ME, USA). All methods were carried out with relevant guidelines and regulations at the University of Alabama at Birmingham (UAB). All zebrafish housing, handling, and tissue collection protocols were approved by the UAB Institutional Animal Care and Use Committee.

### Chronic Unpredictable Stress

**Figure 5:**
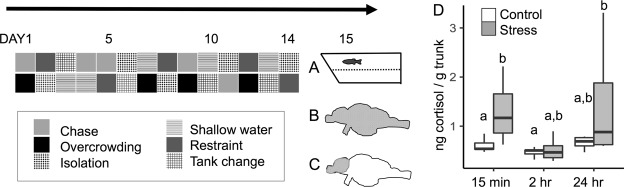
Chronic Unpredictable Stress Experimental Timeline. Animals underwent two different stress experiences each day for 14 days. On day 15, 24 hours after the last stress experience, animals were (**A**) exposed to a novel tank and behavioral responses recorded, (**B**) sacrificed and whole brain fresh frozen for mRNA extraction, or (**C**) sacrificed and telencephalon and olfactory bulb fresh frozen for RNA-seq, or (**D**) Trunk cortisol was taken at one of three time points after the last stress experience. Horizontal lines indicate the median. Vertical lines indicate 1.5 times the interquartile range. Same lower case letters indicate *post hoc* pairwise comparison p > 0.05, different lower case letters p ≤ 0.05. n = 7 for each group.

Modified from Chakravarty *et al*.^11^ and Piato *et al*.^5^, zebrafish experienced two of the following stressors each day, at varying times each day, for 14 days (Fig. 5): overcrowding (fish were confined by mesh dividers in their home tank for 50 minutes), shallow water (fish swam in 1 cm deep water for 2 minutes), social isolation (individuals were confined in 250 ml fish water without visual contact of conspecifics for 50 minutes), object chase (chased with a handle of a net in their home tank for 20 minutes), restraint (individuals were separated by mesh dividers in their home tank so that they touched the dividers when the changed orientation, for 20 minutes), and tank change (netting between two tanks, 8 times). Three cohorts of males (n = 18, n = 24, n = 42) and one of females (n = 24) underwent chronic unpredictable stress or were in the control group. Control animals were not handled during this time.

### Novel Tank Test

Novel tank tests measure anxiety-like behaviors that can be responsive to stress^45,46^. 24 hours after the last stress experience, a subset of one male and female cohort (n = 8, n = 12 respectively) were exposed in a novel 1.5 L tank with fish water for 6 minutes. Swimming behavior was recorded (Sony, Tokyo, Japan). Time spent on bottom half and total swim distance was measured with Ethovision v11 (Noldus, Wageningen, Netherlands).

### Tissue collection, Cortisol measurement, and RNA isolation

Animals were killed by immersion in 4 °C system fish water and tissue was collected and fresh frozen on dry ice < 4 minutes after decapitation. Headless trunks of individuals were immediately frozen on dry ice. Whole brains were collected for a subset of one male and female cohort (n = 10 and = 12, respectively). A separate cohort of males (n = 24) had telencephalon/olfactory bulb and individual optic tectum/hindbrain fresh frozen and collected separately. In this study, we used the region that contained the telencephalon. Four zebrafish telencephalon were pooled per sample, totaling 3 samples for stress and 3 for control groups. All collected tissue was stored at −80 °C.

For a separate cohort of males (n = 42) trunk cortisol levels were assayed at several time points after the last chronic unpredictable stress experience to follow physiological response (Fig. 5). Trunk cortisol extraction slightly modified a previously described protocol^47^. Briefly, headless samples were homogenized in PBS, and cortisol was extracted from the homogenate with diethyl ether after centrifugation at 3500 rpm for 5 minutes, all at 4 °C. The organic layer was isolated in 13 mm × 100 mm glass tubes until the ether evaporated, and samples were reconstituted in 0.5% BSA PBS solution before being tested in duplicate on a commercially available enzyme immunoassay kit (Salimetrics, State College, PA, USA).

Tissue processing for RNA isolation followed the Trizol® manufacture protocol (Sigma-Aldrich, St. Louis, MO, USA). Briefly, frozen tissue was homogenized in Trizol®, then phase separated with chloroform, isolated with glycogen and isopropanol and washed with 75% ethanol, before being resuspended in RNAse free water and incubated at 55 °C. Product was measured for concentration and purity on Nandrop 2000 (Thermo Fisher, Waltham, MA, USA).

### Whole Brain Reverse Transcription Quantitative PCR

RNA samples of 100 ng were treated with AMPD1 DNase I kit, in DNAse l for 15 minutes at room temperature (Sigma-Aldrich). cDNA synthesis followed, using the iScript cDNA synthesis kit according to the manufacturer protocol (Biorad Hercules, CA, USA). Reverse transcription (RT) was followed by quantitative PCR (qPCR), using SsoAdvanced Universal SYBR Green Supermix (Biorad Hercules, CA, USA). Briefly, qPCR parameters were 3 minutes at 95 °C followed by 42 cycles of 10 seconds at 95 °C and 30 seconds at 62.6 °C. This was followed by 1 minutes at 95 °C, and 10 seconds at 55 °C. The comparative Ct method was used^48^; duplicate reactions were run for each primer set and sample, which provided an averaged threshold cycle (CT), from which the reference gene reference elongation factor alpha (*ef1a*) CT was subtracted^49^, giving delta CT (ΔCt). Each ΔCt was normalized to the averaged control delta CT giving ΔΔCt, and a fold change was calculated as 2^−ΔΔCt^. The reference gene *ef1a* expression has been shown to remain unchanged by chemical treatment, age, sex, and has used in previous zebrafish stress studies^18,49^. Primers were designed using NCBI Basic Local Alignment Search Tool PrimerBLAST (www.ncbi.nlm.nih.gov/tools/primer-blast/) to ensure specific gene product, and product was BLASTed to ensure unique matching to gene. RT-qPCR was also used to validate telencephalon RNA sequencing results. Except for primer sequences from previous studies, primers were designed for the following genes: *ache*, *hsd11b2*, *npy*, *elfa*, *loxl2b*, *cdk5*, *chrna7*, *gpx1*, *jun*, *igfbp2a*, *nog2*, *col8a2*, *nub1*, *cayp1*, *ela2l, hey1, her4.2, 6, 8.2*^7,50–52^ (Supplement 3).

### Telencephalon RNA sequencing

Using RNA sequencing we quantified telencephalon and olfactory bulb RNA levels in male adult zebrafish experiencing 14 days of chronic unpredictable stress and control animals. Each group had 3 samples, with 4 pooled telencephalon per sample. To check telencephalon RNA integrity, samples were run through Agilent 2100 Bioanalyzer (Santa Clara, CA, USA) at the UAB Heflin Genomics Core with RNA Integrity Number >8. Telencephalon-isolated RNA was shipped frozen to QuickBiology (Pasadena, CA, USA) for cDNA library preparation according to KAPA Stranded mRNA-Seq polyA selected kit with 201–300 bp KAPA Biosystems (Wilington, MA), using input of 250 ng total RNA. The final library quantity and quality was checked by Agilent 2100 Bioanalyzer and Qubit 2.0 Fluorometer (Life Technologies, Carlsbad, CA, USA). The generated 30 million paired-end reads of 150 bp RNA were sequenced on the Illumina HiSeq 4000 (San Diego, CA, USA)^53–55^.

### Data analysis and statistics

Data were analyzed with the software program R (Vienna, Austria)^56^. For novel tank behavior, and whole brain candidate gene expression, Student’s t-test or nonparametric Wilcoxon rank sum was used. A two-way ANOVA was used for the cortisol assay, and a *post hoc* pairwise t-test with Benjamini-Hochberg corrections was used^57^. Any outliers were excluded. To validate RNA-seq genes expression fold change with RT-qPCR, Pearson product correlation was used.

Bioinformatics analysis was carried out in Galaxy v17.05 (http://usegalaxy.org, Pennsylvania State University, College Station, PA, USA). FastQ Groomer (1.1.1) was used to convert files^58^, and adapter-containing sequences were trimmed using Trim Galore! (v 0.4.3.1). Trimmed fragment quality was checked using FastQC (v 0.69) (www.bioinformatics.babraham.ac.uk/projects/fastqc), and Bowtie2 (v2.3.2.2) to map fragmented cDNA reads to the GRZv10 *Danio rerio* genome (Ensembl Danio_rerio.GRCz10.89.gtf)^59^. Mapped reads for each sample were assembled into transcript-level expression summaries. The summarized data were normalized in concert with the statistical testing of DE, leading to a ranked list of genes with associated *P*-values and fold changes.

Using a bioconductor software package DESeq2 and biomaRt (10.18129/B9.bioc.biomaRt) with false discovery rate (FDR) adjusted p-values < 0.05, we compared the chronic unpredictable stress group to the control^57^.

### Functional annotation of differentially expressed genes

First, genes were individually characterized using the Genecards and Zfin databases to obtain basic information regarding gene function. Gene annotation enrichment analysis was conducted on lists of downregulated and upregulated genes using the DAVID Functional Annotation Tool (version 6.8)^60^, with medium classification stringency and default parameters. Significant functional enrichment was assessed via Benjamini-Hochberg corrected p-values. Putative murine homologs to zebrafish differentially expressed genes were identified using the NCBI Homologene tool. Ontological terms for ORANGE domain containing proteins were obtained via manual search of DAVID database for mouse and zebrafish genes.

RNA-seq data of this study are available in NCBI Sequence Read Archive (SRA) Biosample Database with the accession numbers SRX3503959 and SRX3503960. qPCR and behavior data are available from the corresponding author upon request.

## Supplementary information

Supplement

Supplementary Dataset 1
