# Supplementary material for: Telencephalon transcriptome analysis of chronically stressed adult zebrafish
Source: Sci Rep. 2019 Feb 4;9:1379. doi: 10.1038/s41598-018-37761-7 (PMC6361922; doi:10.1038/s41598-018-37761-7)
Supplement: Supplementary file 1 — Supplement [file 41598_2018_37761_MOESM1_ESM.pdf]

Victoria Huang, Anderson A. Butler, and Farah D. Lubin. Telencephalon transcriptome analysis of chronically stressed adult zebrafish.

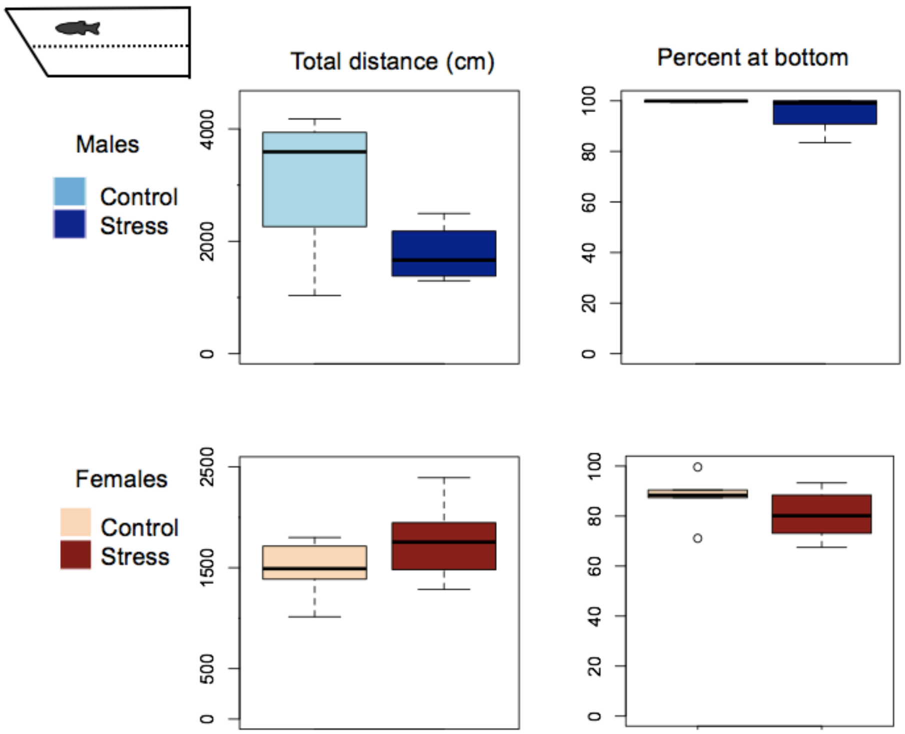

Supplement 1. Novel tank swimming behavior. Total swim distance and percent time at bottom in a novel tank were measured from males and females. Horizontal lines indicate the median. Vertical lines indicate 1.5 times the interquartile range. Open circles indicate values outside the interquartile range. Outliers were not included in anaylsis. Males: N=4 per group. Females: n=6

Supplement 3. Reverse transcription qPCR primer characteristics

Gene	accession	F and R primers 5' -3'	Amplicon length
ache	NM_131846	F GCAAAAGCATGTGGGCTTGA<br>R TCCACTTTCCACTGTCGCTC	147
nr3c1	NM_001020711	F ACAGCTTCTTCCAGCCTCAG<br>R CCGGTGTTCTCCTGTTTGAT<br>*Pavlidis et al 2015	116
hsd11b2	NM_212720	F GCTCCTTTCTCCTCAACCCC<br>R GCTTTATAAGCGCACGAGGC	155
npy	NM_131074	F GGAGGAGCTCGCCAAGTAT<br>R GGGACTCTGTTTCACCAATCA<br>*Drew et al 2008	128
ef1a	NM_131263	F CACCGTTCCAAAGGTTGCG<br>R TGGCAACAGGTGCAGTTCTAA	224
loxl2b	NM_001080626	F TGTGAAATTTGCACTCGCAGC<br>R TTCAGGCTCCTGGTAGGCTAT	156
cdk5	NM_131719	F AGGCCTCCGGATGTACTGTT<br>R TCGTCCACATCGTTACCAGG	128
chrna7	NM_201219	F GCCGTACCCAGATGTGACTT<br>R GCAGGGCCAGAGTAGAGATG	104
gpx1a	NM_001007281	F ACGACCCTGTGTCCCTTAT<br>R CTTCTGCTGTACCTCTTGAATG<br>*Guan et al 2011	133
jun	NM_199987	F CCGACGTGGGACTTCTCAAA<br>R ATCCGTCACGTTCTTGGGAC	125
igf1bp2a	NM_131458	F GACCCTAAAGCACCACATGC<br>R GAGATCCTTTCCAGCACCTG<br>* Arslan-ergul 2014	71
acyp1	NM_001122849	F ACGAGCTGTTGTCTGTGGATT<br>R CGGTTCCAGCATCAGTGTTC	129
col8a2	NM_001082901	F TGCCAAGACATTCCCGAGAC<br>R AACTTCTGCAGGTCAGGTGG	125
ela2l	NM_199886	F GGTGTGGATGTGCGTCCTAA<br>R CAGTGAGCGGCAGTCAAAAC	128
nog2	NM_130992	F GCCTCACCTGAGCGAGTATC<br>R GCGCCCACGTGCATAAATAG	135
nub1	NM_001113580	F CCCTACCTTACCTGGTGCTGG<br>R AGCACCGCATAATTGTCCAC	85
hey1	NM_212561.1	F CTGAGGATACGCGCTGCTAA<br>R GATGATCCCTCTGCGACGTT	199
her6	NM_131079.2	F TGAACTCGGGACACTTCGTG<br>R GCACAGCTGCTTCTAGTGGA	91
her4.2	NM_131090.4	F AGCTTTCTGTCTCAGTGCCC<br>R GATGTGACTGTGGGCTGGAG	171
her8.2	NM_001166166.1	F GAGCAGTAGAGTTTCTGACCCT<br>R AAGCGGGATCCAAGGGTTTT	138
